# The role of tunable nonlinear dark resonances on vacuum Rabi splitting and optical bistability in an atom-cavity system

**DOI:** 10.1038/s41598-021-89652-z

**Published:** 2021-05-18

**Authors:** SiJia Hui, Feng Wen, Minghui Zhang, ShaoWei Zhang, YuanJie Yang, ZhiPing Dai, YungPeng Su, YanPeng Zhang, HongXing Wang

**Affiliations:** 1 Key Laboratory for Physical Electronics and Devices of the Ministry of Education & School of Science & Shaanxi Key Lab of Information Photonic Technique & Institute of Wide Bandgap Semiconductors, Xi’an Jiaotong University, Xi’an, 710049 China; 2 School of Physics, University of Electronic Science and Technology of China, Chengdu, 611731 China; 3 College of Physics and Electronic Engineering, Hengyang Normal University, Hengyang, 421002 China; 4 State-owned Sida Machinery Manufacturing, Xianyang, 712201 China

**Keywords:** Nonlinear optics, Software

## Abstract

The phenomenon of “dark resonances” is a well-known concept in quantum optics and laser spectroscopy. As a general rule, interactions involving in such a “dark state” lead to multiple quantum superposition states that interact coherently and are undesirable. In this paper, two types nonlinear interaction in an atomic cavity, namely the nested and cascaded interactions, are theoretically analyzed how the dark resonances form the dark state peak to modulate the vacuum Rabi splitting (VRS) and optical bistability (OB) behavior. In both the zero- and high order modes, there are four VRS peaks generated in the nested interaction and three in the cascade interaction. Dark resonance can modulate not only the peak number of VRS, but also the OB thresholds. It is found that dark state can determine the asymmetric OB distribution of nested type and symmetric OB distribution of cascade type. Besides that, the distinctive OB thresholds in two kinds of interaction also be studied. The observations not only conceptually extend the conventional “dark resonances” phenomenon, but also opens the door for a variety of new applications in tunable all-optical switch and quantum communication.

## Introduction

The wide application of dressed theory^1^ and electromagnetically induced transparency (EIT)^2–4^ for dark state generation in a nonlinear medium have triggered innovative works towards effectively modulating^5, 6^ the output intensity^7, 8^. As a kind of dressed effect, the past decade has seen the rapid development of vacuum Rabi splitting (VRS) in modulating the strongly coupled signal of a two-level cavity-atom system with coupling strength *g*^9–13^. For instance, at the condition of $g > \kappa ,\gamma$, where $\kappa ,\gamma$ refer to the cavity decay rate and atomic decay rate, the transmission peak splits into two for the dressed effect. And the interval between the split energy levels is $2g\sqrt N$, where *N* is the number of excited atoms^14^. A lot of work has been concentrated on VRS of two-level atom system (normal-mode splitting), such as in atomic beams^15^, cold atomic cloud^16, 17^, and a Bose–Einstein condensate^18–20^. Specifically, the normal-mode splitting in case of $g\sqrt N \ll \Delta_{FSR}$, corresponding to free spectral region, have been reported widely, which is equated with generation a single cavity resonant mode splitting close to the atomic resonance. In recent years, there has been an increasing interest in modulating the super coupling strength $g\sqrt N > \Delta_{FSR}$ by increasing the number of excited atoms or the strength of the atom-cavity *g* to reach the super strong coupling, such as the super coupling strength in cavity quantum electrodynamics (cavity QED). Although considerable efforts have been put into VRS in two-level structure, the physics and principle of the multi-level structure are still unclear. Therefore, this study will develop the VRS into coherent multilevel atoms system and present a kind of multi-order mode appear in the splitting transmission spectra.

Inspired by the preparation of VRS, optical bistable (OB) modulation has also been experimentally observed in the cavity-atom system. A classic example is that both the quantum correlated bright light beams and controllable multistability^21^ are obtained in a coherent atom setting in optical parametric oscillator (OPO). It has been conclusively noted that the OB rising from feedback effect depends on the real excitation states of the material^22–24^. Hitherto, few studies have investigated the OB behavior under the effect of nonlinear interaction process. Therefore, we can combine multi-mode VRS and OB in the ring cavity-atom cavity, and then use different nonlinear interactions to study the diverse effects on VRS and OB. Because the dark state plays an important role in the modulation of VRS and OB, our research model can be applied to many areas which dark state are limited, such as data storage^25, 26^, slow light^27^, and quantum teleportation^28, 29^.

The aim of this paper is to explore how the nested interaction and cascaded interaction affect the VRS and OB behavior in a cavity-atom coupled system. By employing the dressed effect theory, we show that the nested three photon resonance can produce indirect dressed effect to split the signal to four VRS peaks. While dual two-photon resonances of cascade type produce dark resonance to split signal into three VRS. peaks. Since the photon dark state is a decisive factor of FWM (four wave mixing) peaks splitting in the multi-order modes, the modulation of not only VRS but also OB behavior can be realized by adjusting the nested and cascaded interactions. As a result, asymmetric distribution of three intensity peaks and two points of no OB behaviors can be generated in the nested type, while four intensity peaks and two points of no OB of symmetric distribution can be generated in the cascade type. In particular, the continuous extended right OB threshold and the constant left OB threshold are found in the nested type, which contrasts with the simultaneously increased left and right thresholds in the cascaded type.

The rest of this paper is organized as following. In Section II, we introduce the setup to realize the splitting FWM signal that adopts two kinds of interactions: the nested type and the cascaded type. In Section III, we investigate the multi-dressed VRS and study how to modulate the dark and bright states. In Section IV, we present the VRS of high-order modes transmission spectra with the distinguished adjustable FWM peaks. In Section V, we explore how the OB distribution and scope can be effectively modulated by the nested and cascaded interaction. In Section VI, we make a summary of this study.

## Basic theory

**Figure 1:**
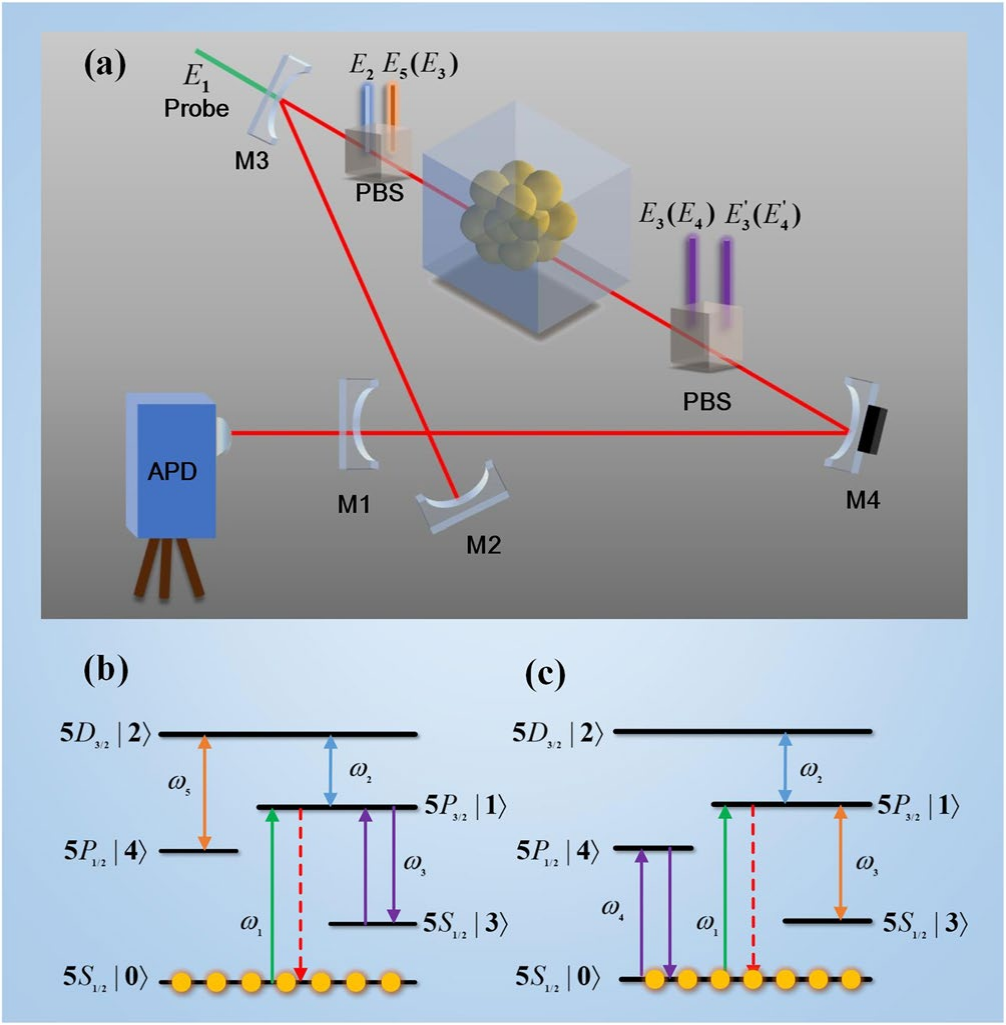
(**a**) Diagrammatic setup of FWM signal generation, consisting of four reflectors to form the ring cavity, and embedded nonlinear media ^85^Rb to produce the signal. The cavity frequency can be modulated by the piezoelectric transducer (PZT) that is mounted on mirror M4. An avalanche photodiode (APD) is placed at the terminal of the setup to detect the signal. PBS is the piezoelectric beam splitter. (**b**) The nested type interaction mode coupled with five energy levels. (**c**) The cascade type interaction mode coupled with five energy levels.

The tunable FWM signals under different dressed effects are carried out in a cavity-atom coupled system. The ring cavity consists of a half-transmission t1 (t3) and two half-reflection r1 (r3) mirrors (M1 and M3) and two high-quality reflectors (M2 and M4), all of which satisfy the condition $r_{i}^{2} + t_{i}^{2} = 1(i = 1,3)$. The cavity frequency can be tuned by a piezoelectric transducer (PZT) which is attached to M4. We control the length of the cavity Lc at $L_{c} = 20\;{\text{cm}}$ to meet the phase interference condition $\Delta \varphi = \frac{2n\pi }{\lambda }2L = q2\pi (q = 0,1,2...)$ and realize multi-order longitudinal modes in the cavity. Besides, in order to reduce the window loss of the beam and encapsulate atomic to observe the interaction between light and medium, a Brewster window of $L_{a} = {1}0\;{\text{cm}}$ is designed in the cavity window. On the one hand, it can shape certain polarization directions for the purpose of high accuracy of experiment. On the other hand, it is to ensure that the external field will not generate noise in the cavity resonance. On completing the cavity, the atom ensemble ^85^Rb is placed in cavity to form the cavity-atom coupled system which showed in Fig. 1(a). The system contains five energy levels: a ground state $|0\rangle$ and four excited states $|1\rangle$, $|{2}\rangle$, $|{3}\rangle$, and $|{4}\rangle$.

In order to understand how nonlinear interaction regulates FWM signal, two kinds of interaction named as nested type and cascaded type are performed. For nested interaction, a horizontally polarized probe field *E*_1_ coupled with energy level transition $|0\rangle \to |1\rangle$ and another two pairs laser *E*_3_ and $E_{3}^{{\prime}}$ drive the energy level transition $|3\rangle \to |1\rangle$. Once satisfying the law of conservation of momentum ($k_{Fn} = k_{3} + k_{3}^{{\prime}} - k_{1}$), the FWM signal will generate. Simultaneously, additional coupling fields (*E*_2_ and *E*_5_) are injected into the nonlinear medium to couple with transition energy levels $|1\rangle \to |2\rangle$ and $|5\rangle \to |2\rangle$, respectively. Following this process, the nested dressed FWM signal is produced and shows in the energy level diagram of Fig. 1b. For cascaded interaction, probe filed *E*_1_ couple with transition energy level $|0\rangle \to |1\rangle$ and *E*_4_ with $E_{4}^{{\prime}}$ couple with another energy level transition $|0\rangle \to |4\rangle$ to generate the FWM signal which also obey the law of conservation of momentum $k_{Fc} = k{}_{4} + k_{4}^{{\prime}} - k_{1}$. When cascade dressed fields *E*_2_ and *E*_3_ couple with level transition $|2\rangle \to |1\rangle$ and $|3\rangle \to |1\rangle$, respectively, the cascaded dressed FWM signal will generate and the energy level diagram performed in Fig. 1c. It should be noted that the we set the two pairs coupling lasers *E*_3_ and $E_{{3}}^{{\prime}}$ (*E*_4_ and $E_{4}^{{\prime}}$) propagating in the reverse direction of *E*_1_ in order to eliminate the Doppler effects. The density matrix equations and perturbation chain of the two types interaction are employed to simulate the FWM signals variation. For the nested interaction, the perturbation chain is $\rho_{00}^{(0)} \mathop{\longrightarrow}\limits^{{\omega_{1} }}\rho_{{(G_{2} \pm_{{G_{5} \pm }} )0}}^{(1)} \mathop{\longrightarrow}\limits^{{\omega_{3} }}\rho_{30}^{(2)} \mathop{\longrightarrow}\limits^{{ - \omega_{3} }}\rho_{10}^{(4)}$ and the equation for the third-order density element of the nested interaction as follows:

1 $$\rho_{Fn}^{(3)} = \frac{{ - iG_{{{\text{Fn}}}} }}{{\left( {d_{1} + \left| {G_{2} } \right|^{2} /[d_{2} + {{\left| {G_{5} } \right|^{2} } \mathord{\left/ {\vphantom {{\left| {G_{5} } \right|^{2} } {d_{5} }}} \right. \kern-\nulldelimiterspace} {d_{5} }}]} \right)^{2} d_{3} }}$$

In the Eq. (1), $G_{{{\text{Fn}}}} = G_{1} G_{3} \left( {G^{\prime}_{3} } \right)^{*} \exp (i{\mathbf{k}}_{{{\text{Fn}}}} \cdot {\mathbf{r}})$ and the Rabi frequency for the field $E_{{1}}$, $E_{3}$ and $E_{3}^{{\prime}}$ are $G_{{1}}$, $G_{3}$, and $G_{3}^{{\prime}}$, respectively. For the cascade type, the perturbation chain is $\rho_{00}^{(0)} \mathop{\longrightarrow}\limits^{{\omega_{4} }}\rho_{40}^{(1)} \mathop{\longrightarrow}\limits^{{ - \omega_{4} }}\rho_{00}^{(2)} \mathop{\longrightarrow}\limits^{{\omega_{1} }}\rho_{{(G_{{2}} \pm G_{3} \pm )0}}^{(4)}$ and the equation for the third-order density element of the nested type is

2 $$\rho_{Fc}^{(3)} = \frac{{ - iG_{{{\text{Fc}}}} }}{{\left( {d_{1} + \left| {G_{2} } \right|^{2} /d_{2} + {{\left| {G_{3} } \right|^{2} } \mathord{\left/ {\vphantom {{\left| {G_{3} } \right|^{2} } {d_{3} }}} \right. \kern-\nulldelimiterspace} {d_{3} }}} \right)^{2} d_{4} }},$$

where $G_{{{\text{Fc}}}} = G_{1} G_{4} \left( {G^{\prime}_{4} } \right)^{*} \exp (i{\mathbf{k}}_{{{\text{Fc}}}} \cdot {\mathbf{r}})$. $G_{4}$ and $G_{4}^{{\prime}}$ are the Rabi frequency for the field $E_{{4}}$ and $E_{{4}}^{{\prime}}$, respectively. The other parameters are $d_{1} = \Gamma_{10} + i\Delta_{1}$, $d_{2} = \Gamma_{20} + i\left( {\Delta_{1} + \Delta_{2} } \right)$, $d_{3} = \Gamma_{30} + i\left( {\Delta_{1} - \Delta_{3} } \right)$, $d_{4} = \Gamma_{14} + i\left( {\Delta_{1} - \Delta_{4} } \right)$, and $d_{5} = \Gamma_{40} + i\left( {\Delta_{1} + \Delta_{2} - \Delta_{5} } \right)$. $\Gamma_{ij}$ is the transverse relaxation rate between the state $|i\rangle$ and $|j\rangle$. $\Delta_{i} = \Omega_{i} - \omega_{i}$ is the frequency detuning, corresponding to the difference between the resonance frequency $\Omega_{i}$ and pump frequency $\omega_{i}$. As the cavity field is another dressed field that influences the FWM signal, we modify the density element Eqs. (1) and (2) by adding the coupling strength $g\sqrt N$, which couple to level transition $|0\rangle \to |1\rangle$. If the system is in an equilibrium state, the expression of the nested type FWM cavity mode is transformed to

3 $$a_{Fn} = \frac{{ - ig\sqrt N G_{{{\text{Fn}}}} }}{{\left( {d_{1} + \left| {G_{2} } \right|^{2} /[d_{2} + {{\left| {G_{5} } \right|^{2} } \mathord{\left/ {\vphantom {{\left| {G_{5} } \right|^{2} } {d_{5} }}} \right. \kern-\nulldelimiterspace} {d_{5} }}]} \right)d{}_{1}^{{\prime}} }}.$$

For the cascade type, the FWM cavity mode is

4 $$a_{Fc} = \frac{{ - ig\sqrt N G_{{{\text{Fc}}}} }}{{\left( {d_{1} + \left| {G_{2} } \right|^{2} /d_{2} + {{\left| {G_{3} } \right|^{2} } \mathord{\left/ {\vphantom {{\left| {G_{3} } \right|^{2} } {d_{3} }}} \right. \kern-\nulldelimiterspace} {d_{3} }}} \right)d{}_{1}^{{\prime}} }},$$

with $$d_{1}^{{\prime}} = \gamma + i(\Delta_{1} - \Delta_{ac} )$$.

## Modulation of the dressed VRS

A comparative simulation is adopted to study the modulation of VRS by nested and cascaded interaction. Using the different mechanism of the nested and cascaded interactions, we investigated the splitting numbers and positions modulation of zero-mode FWM signals. And then we use the dressed theory to verify the different effects of nested and cascaded types on FWM signals.

### Multi-dressed effects on VRS

**Figure 2:**
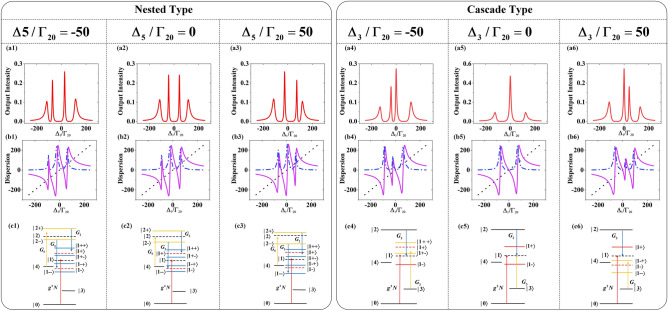
(**a1**)–(**a3**) The nested FWM signal spectra when scan $\Delta_{1}$. (a1) With $\Delta_{5} /\Gamma_{20} = { - }50$. (**a2**) With $\Delta_{5} /\Gamma_{20} = 0$ (a3) With $\Delta_{5} /\Gamma_{20} = 50$. (**b1**)–(**b3**) Intracavity dispersion curves (solid curves), absorption curves (dashed curves), and detuning $\Delta_{1}$ lines (dotted lines), corresponding to (**a1**)–(**a3**). (**c1**)–(**c3**) The dressed five-energy levels diagram, corresponding to (**a1**)–(**a3**). (**a4**)–(**a6**) The cascaded FWM signal spectra when scan $\Delta_{1}$. (**a4**) With $\Delta_{3} /\Gamma {}_{20} = { - }50$ (**a5**) With $\Delta_{3} /\Gamma_{20} = 0$. (**a6**) With $\Delta_{3} /\Gamma_{20} = 50$. (**b4**)–(**b6**) Intracavity dispersion and absorption curves corresponding to (**a4**)–(**a6**). (**c4**)–(**c6**) The dressed five-energy levels diagram, corresponding to (**a1**)–(**a4**). The other parameters are $G_{2} = 50\,{\text{MHz}}$, $G_{3} = 50\,{\text{MHz}}$, $G_{5} = 50\,{\text{MHz}}$, $\Delta_{2} = 0\,{\text{MHz}}$ and $\Delta_{ac} = 0\,{\text{MHz}}$.

The first step in this process is to investigate the FWM signal modulation under the nested interaction. As shown in Fig. 2(a1), (a2) and (a3), they present four splitting FWM peaks and peak positions can be changed by controlling single factor variable $\Delta_{5} /\Gamma_{20} = - 50,0,50$, which induced by $E_{5}$. Once cavity field $g^{2} N$ coupled with level $|1\rangle$, the dressed states $|1 \pm \rangle$ are generated and the two eigenvalues are $\lambda_{1 \pm } = \Delta_{ac} /2 \pm \sqrt {\Delta_{ac}^{2} /4 + g^{2} N}$. Simultaneously, when $E_{5}$ is strong enough to couple with upper level ${|2}\rangle$, dressed states ${|2} \pm \rangle$ are generated with eigenvalues $\lambda_{{{2} \pm }} = \Delta_{5} /2 \pm \sqrt {\Delta_{5}^{2} /4 + \left| {G_{5} } \right|^{2} }$. One significant property of the dressed states ${|2} \pm \rangle$ is that they can provide two transition channels for field $E_{{2}}$ to couple with ${|2 + }\rangle \to |{1}\rangle$ and ${|2} - \rangle \to |{1}\rangle$. When $E_{5}$ dressed on level ${|2}\rangle$, followed by $E_{{2}}$ deriving the secondary dressed effect on dressed state ${|1 + }\rangle$ and ${|1} - \rangle$, the nested three photon indirect dressed effect is realized. The indirect dressed effect defined as $E_{5}$ cannot work on the energy level ${|1}\rangle$, but can dressed on the upper level ${|2}\rangle$. On the one hand, secondary dressed effect can split ${|1 + }\rangle$ into ${|1 + } \pm \rangle$ and eigenvalues are $\lambda_{{{1 + } \pm }} = (\Delta_{{2}} + \lambda_{{{2} \pm }} - \lambda_{{1 + }} {)/2} \pm \sqrt {(\Delta_{2} + \lambda_{{{2} \pm }} - \lambda_{{1 + }} {)}^{2} {/4 + }\left| {G_{2} } \right|^{2} }$. On the other hand, dressed state ${|1} - \rangle$ can also be secondary split into states ${|1} - \pm \rangle$ and the eigenvalues are $\lambda_{{1{ - } \pm }} = (\Delta_{{2}} + \lambda_{{{2} \pm }} - \lambda_{{1{ - }}} {)/2} \pm \sqrt {(\Delta_{2} + \lambda_{{{2} \pm }} - \lambda_{{1{ - }}} {)}^{2} {/4 + }\left| {G_{2} } \right|^{2} }$. When probe field $E_{{1}}$ satisfies the four bright state conditions $\Delta {}_{{1}}{ + }\lambda_{{1 + }} { + }\lambda_{{1 + + }} = 0$, $\Delta {}_{{1}}{ + }\lambda_{{1 + }} { + }\lambda_{{1 + - }} = 0$, $\Delta {}_{{1}}{ + }\lambda_{{1{ - }}} { + }\lambda_{{1{ - + }}} = 0$ and $\Delta {}_{1} + \lambda_{1 - } + \lambda_{1 - } = 0$, four splitting FWM peaks are obtained. These four secondary dressed energy states, corresponding to the four eigenvalues, depicted in Fig. 2(c1), (c2) and (c3), which consistent with the four VRS peaks in Fig. 2(a1), (a2) and (a3). After clarifying that VRS peak will be split into four under nested interaction, we will discuss the positions changes of these peaks. The four FWM peaks shifting toward the negative region of scanning $\Delta_{1}$ in Fig. 2(a1), as the consequence of dressed states $|2 \pm \rangle$ shifts up at condition $\Delta_{5} /\Gamma_{20} = - 50$ which depicted in Fig. 2(c1). In Fig. 2(a2), these four peaks distributed symmetrically at center $\Delta_{1} = 0\;{\text{MHz}}$ because $E_{5}$ resonantly coupled with level $|2\rangle$ and dressed states $|2 \pm \rangle$ symmetrically distribute with level $|2\rangle$ as shown in Fig. 2(c2). At the case of $\Delta_{{5}} {/}\Gamma_{{{20}}} = 50$, Fig. 2(a3) presents that four peaks shift to the positive region of scanning $\Delta_{1}$ for dressed stated $|2 \pm \rangle$ shifts down, which exhibited in Fig. 2(c3). In addition, we observed that the intensity height of four peaks would also change, because the closer the dressed state is to the intrinsic energy level, the lower the dispersion will be, resulting in less energy reduction. Further, the effects of absorption and dispersion^30^ in the FWM signal are investigated. The results observed from Fig. 2(b1), (b2) and (b3) identify that the intersecting points of the detuning line and dispersion curve represent the FWM signal peaks and the disappearance peaks at the middle intersection of FWM spectra. The dark resonance, which results in strong absorption and disappearance of dispersion, is the dominant factor to decide the disappearance peaks at the middle intersection of FWM spectra.

Secondly, the splitting FWM signal of the cascade type is under discussed. Different from the nested interaction, Fig. 2(a4), (a5) and (a6) exhibit the number of VRS peaks can be four or three by controlling single factor variable $\Delta_{{3}} {/}\Gamma_{{{20}}} = - 50,0,50$, which $E_{3}$ induced. After $E_{2}$ resonantly dresses the virtual level ${|1}\rangle$ and $E_{{3}}$ selectively couples with the dressed state ${|1} + \rangle$ or $|1 - \rangle$ or virtual level ${|1}\rangle$, the cascaded dual two-photon direct dressed effect will achieve. The dressed state ${|1 + }\rangle$ will split into secondary dressed states ${|1 + } \pm \rangle$ at $\Delta_{3} /\Gamma_{20} = - 50$ and the eigenvalues are $\lambda_{1 + \pm } = - (\Delta_{3} + \lambda_{1 + } )/2 \pm \sqrt {(\Delta_{3} + \lambda_{1 + } )^{2} /4 + \left| {G_{3} } \right|^{2} }$. $E_{3}$ will interact with virtual level ${|1}\rangle$ under condition $\Delta_{3} /\Gamma_{20} = 0$, which will cause dark state resonance. The dressed state $|1 - \rangle$ will split into secondary dressed states $|1 - \pm \rangle$ at $\Delta_{3} /\Gamma_{20} = 50$ and the eigenvalues are $\lambda_{1 - \pm } = - (\Delta_{3} + \lambda_{1 - } )/2 \pm \sqrt {(\Delta_{3} + \lambda_{1 - } )^{2} /4 + \left| {G_{3} } \right|^{2} }$. Four VRS peaks are displayed in Fig. 2(a4) at $\Delta_{3} /\Gamma_{20} = - 50\;{\text{}}$, where three peaks generated when probe field $E_{{1}}$ satisfies three bright state conditions $\Delta {}_{{1}}{ + }\lambda_{{1 + }} { + }\lambda_{1 + + } = 0$, $\Delta {}_{{1}}{ + }\lambda_{{1 + }} { + }\lambda_{{1 + { - }}} = 0$ and $\Delta {}_{{1}}{ + }\lambda_{{1{ - }}} = 0$ while another distinct dark-state peak origin from probe field $E_{{1}}$ satisfying the dark resonance condition $\Delta_{{1}} { + }\Delta_{{2}} = 0$. Figure 2(a4) shows the position modulation of FWM peaks shifting toward the negative region of scanning $\Delta_{1}$ when $E_{3}$ works on the upper dressed state $|1 + \rangle$. The three dressed energy levels and a virtual level depicted in Fig. 2(c4), corresponding to the three FWM peaks and a dark-state peak. Figure 2(a5) shows that there are three VRS peaks at condition $\Delta_{{3}} {/}\Gamma_{{{20}}} = 0$, among which the dark state peak of super high intensity is caused by the dual two-photon dark resonances. Once $E_{2}$ and $E_{3}$ simultaneously resonate with virtual level ${|1}\rangle$, double dark states are realized when probe field satisfies the two-photon resonance conditions $\Delta_{{1}} { + }\Delta_{{2}} = 0$ and $\Delta_{{1}} - \Delta_{3} = 0$ in the same time. The other two side peaks appear when probe field $E_{1}$ satisfies two bright state conditions $\Delta {}_{{1}}{ + }\lambda_{{1 + }} = 0$ and $\Delta {}_{{1}}{ + }\lambda_{{1{ - }}} = 0$. Two sided peaks homogeneously distribution with dark state peak as the center, which equivalent to level $|1\rangle$ symmetrically split into dressed states $|1 \pm \rangle$ in Fig. 2(c5). Four VRS peaks performed in Fig. 2(a6) at condition $\Delta_{3} /\Gamma_{20} = 50$, among which three peaks satisfy the bright state conditions $\Delta {}_{{1}}{ + }\lambda_{{1 - }} { + }\lambda_{{1{ - } + }} = 0$, $\Delta {}_{{1}}{ + }\lambda_{{1 - }} { + }\lambda_{{1{ - - }}} = 0$ and $\Delta {}_{{1}}{ + }\lambda_{{1{ + }}} = 0$ while center dark state peak satisfies the dark resonance condition $\Delta_{{1}} { + }\Delta_{{2}} = 0$. The FWM peaks shifting toward the positive region of scanning $\Delta_{1}$ as $E_{3}$ works on the lower dressed state $|1 - \rangle$ and the diagram of splitting dressed state $|1 - \rangle$ is depicted in Fig. 2(c6). Moreover, the dark state peak, corresponding to zero absorption point in Fig. 2(b4), (b5) and (b6), suggested that two-photon dark resonance suppress the absorption, which plays a completely different role in the nested and cascaded interactions. Besides, the changeable peaks intensity can be realized by tuning coupling field detuning in nested and cascade interactions, corresponding to control the width of EIT windows. In Fig. 2 (c), when detuning $\left| {\Delta_{5} } \right|$ and $\left| {\Delta_{3} } \right|$ increase from 0 MHz, the EIT windows distributed asymmetrically and leaded to the narrowed and enlarged EIT windows. When probe filed coupled in the narrowed width of EIT windows, the VRS peaks with relative high intensity will generated. On the contrary, when probe filed coupled in the enlarge width of EIT windows, the VRS peaks with relative low intensity will produce.

### The relationship of detuning parameters with VRS

**Figure 3:**
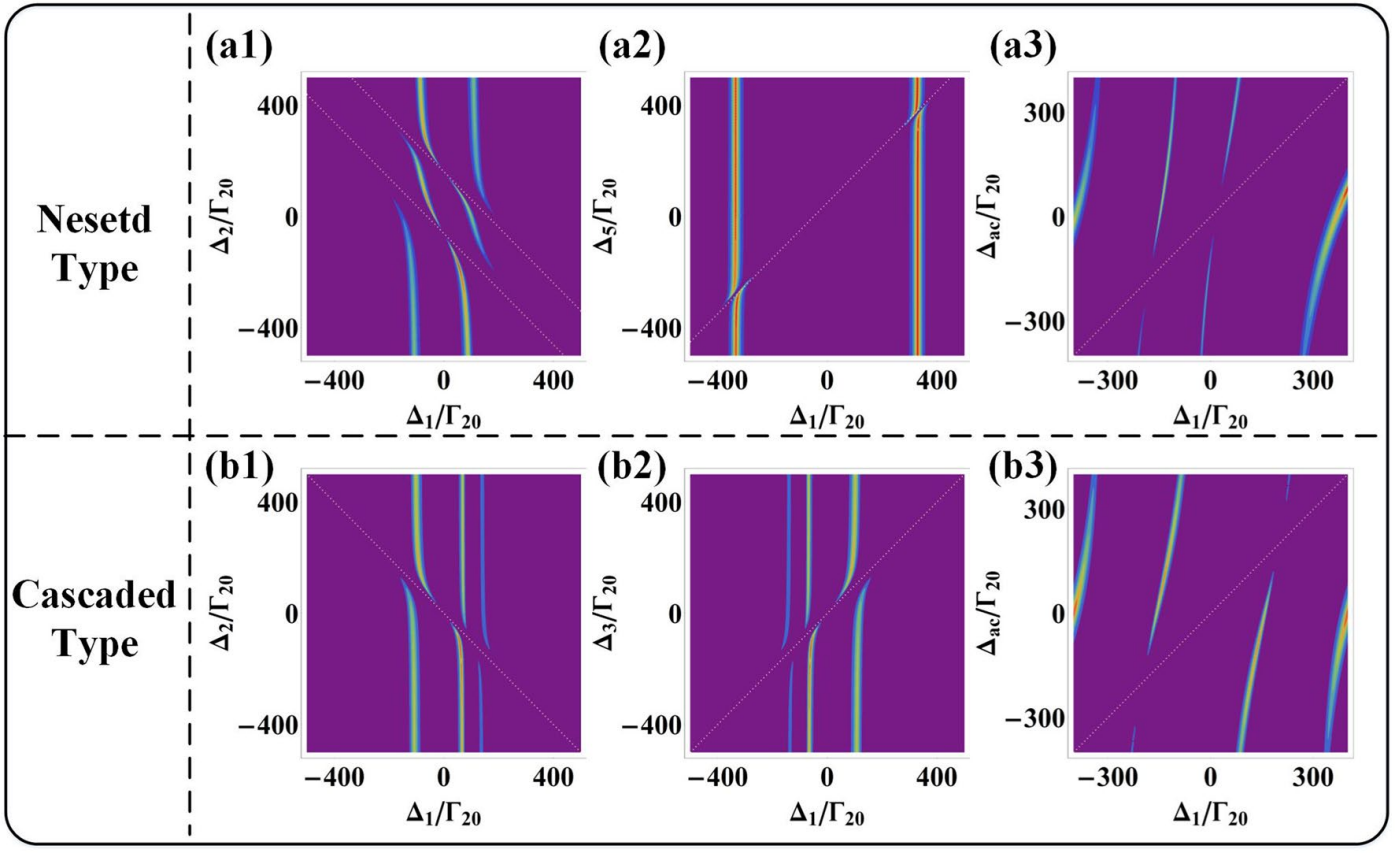
(**a1**) The interrelationship as function of $\Delta_{1}$ and $\Delta_{2}$ at $\Delta_{5} /\Gamma_{20} = 100$. (**a2**) The interrelationship as function of $\Delta_{1}$ and $\Delta_{5}$ at $\Delta_{2} /\Gamma_{20} = 50$. (**a3**) The interrelationship as function of $\Delta_{1}$ and $\Delta_{ac}$ at $\Delta_{2} /\Gamma_{20} = 80$ and $\Delta_{5} /\Gamma_{20} = 0$. (**b1**) The interrelationship as function of $\Delta_{1}$ and $\Delta_{2}$ at $\Delta_{3} /\Gamma_{20} = 100$. (**b2**) The interrelationship as function of $\Delta_{1}$ and $\Delta_{3}$ at $\Delta_{2} /\Gamma_{20} = 100$. (**b3**) The interrelationship as function of $\Delta_{1}$ and $\Delta_{ac}$ at $\Delta_{2} /\Gamma_{20} = 200$ and $\Delta_{3} /\Gamma_{20} = 200$.

Having discussed how the two kinds of interaction modulate the FWM signal, we will study the influence of probe field and other coupling fields on VRS at the same time, corresponding to the modulation effect of additional fields. Figure 3 demonstrates VRS peaks suppressed by different two-photon resonance conditions as show as the splitting peaks dashed lines. Figure 3(a1) shows that FWM peaks are segmented by two dashed lines when probe field satisfies two dark resonance conditions $\Delta_{1} + \Delta_{2} - \lambda_{2 + } = 0$ and $\Delta_{1} + \Delta_{2} - \lambda_{{2{ - }}} = 0$, as the consequence of three-photon indirect interaction that *E*_5_ splits upper level $|2\rangle$ into dressed states $|2 \pm \rangle$ and detuning $\Delta_{2}$ induced by *E*_2_ change to be $\Delta_{2} - \lambda_{2 \pm }$. When scan $\Delta_{1}$ and $\Delta_{5}$, the position to splitting peaks can be changed by tuning different $\Delta_{2}$ to satisfy dark resonance condition $\Delta_{1} + \Delta_{2} - \Delta_{5} = 0$. By setting $\Delta_{2} /\Gamma_{20} = 50$, the up offset splitting peaks lines in Fig. 3(a2) is realized. Figure 3(a3) shows the coupling relation between probe field and cavity field with three bending avoided-crossing plots and dashed lines to split the peaks satisfied the two-photon resonance condition $\Delta {}_{1} - \Delta {}_{ac} = 0$. Different from the nested type, only one line to split peaks line can be generated in the cascade type, because the two-photon interaction cannot dress the upper level ${|2}\rangle$. Figure 3(b1) displays the VRS peaks divided when scan $\Delta_{1}$ and $\Delta_{2}$ and satisfies the two-photon resonance condition $\Delta_{1} + \Delta_{2} = 0$. Similarly, the splitting VRS peaks line and the avoided-crossing plots shown in Fig. 3(b2) and (b3) can be obtained when probe field satisfies the dark resonant conditions $\Delta_{1} - \Delta_{3} = 0$ and $\Delta_{1} - \Delta_{ac} = 0$, respectively.

## Dressed effects of high-order modes on VRS

In this section, we will study the modulation of VRS peak number and position changes of high-order mode VRS both in nested and cascaded interactions. Considering the influence of the cavity field on the transmission signal of FWM, we use transmission coefficient with the dressed effect of the cavity field $g^{2} N$ to modify the expression of the cavity transmission coefficient of FWM signal^31–33^:

5 $$T = \frac{{(t_{3} t_{1} )^{2} e^{{ - \alpha L_{a} }} }}{{[(1 - r_{3} r_{1} e^{{ - \alpha L_{a} /2}} )^{2} + 4r_{3} r_{1} e^{{ - \alpha L_{a} /2}} \sin^{2} (\phi /2)]}},$$

where $\phi (\omega_{F} ) = 2\pi (\Delta_{ac} - \Delta_{1} )/\Delta_{FSR} + (n - 1)L_{a} \omega_{F} /c$ is the ring-cavity phase shift. $\Delta_{FSR} = 2\pi /(L_{c} /c)$ is the free spectral range (FSR) of the empty optical cavity and *c* is the light speed in vacuum. The absorption coefficient and reflective index of the susceptibility $\chi$ are $\alpha = 2(\omega_{F} /c){\text{Im}} [(1 + \chi )^{1/2} ]$ and $n = {\text{Re}} [(1 + \chi )^{1/2} ]$, respectively. The susceptibilities for the nested type $\chi_{n}$ and the cascade type $\chi_{c}$ are expressed as follows:

6 $$\chi_{n} = \frac{{2g^{2} NL_{c} }}{{L_{a} \omega_{F} }} \times \frac{i}{{d_{1} + \left| {G_{2} } \right|^{2} /[d_{2} + (\left| {G_{5} } \right|^{2} /d_{5} )]}},$$

7 $$\chi_{n} = \frac{{2g^{2} NL_{c} }}{{L_{a} \omega_{F} }} \times \frac{i}{{d_{1} + \left| {G_{2} } \right|^{2} /d_{2} + \left| {G_{3} } \right|^{2} /d_{3} )}}.$$

### Splitting of high-order modes in the nested type

**Figure 4:**
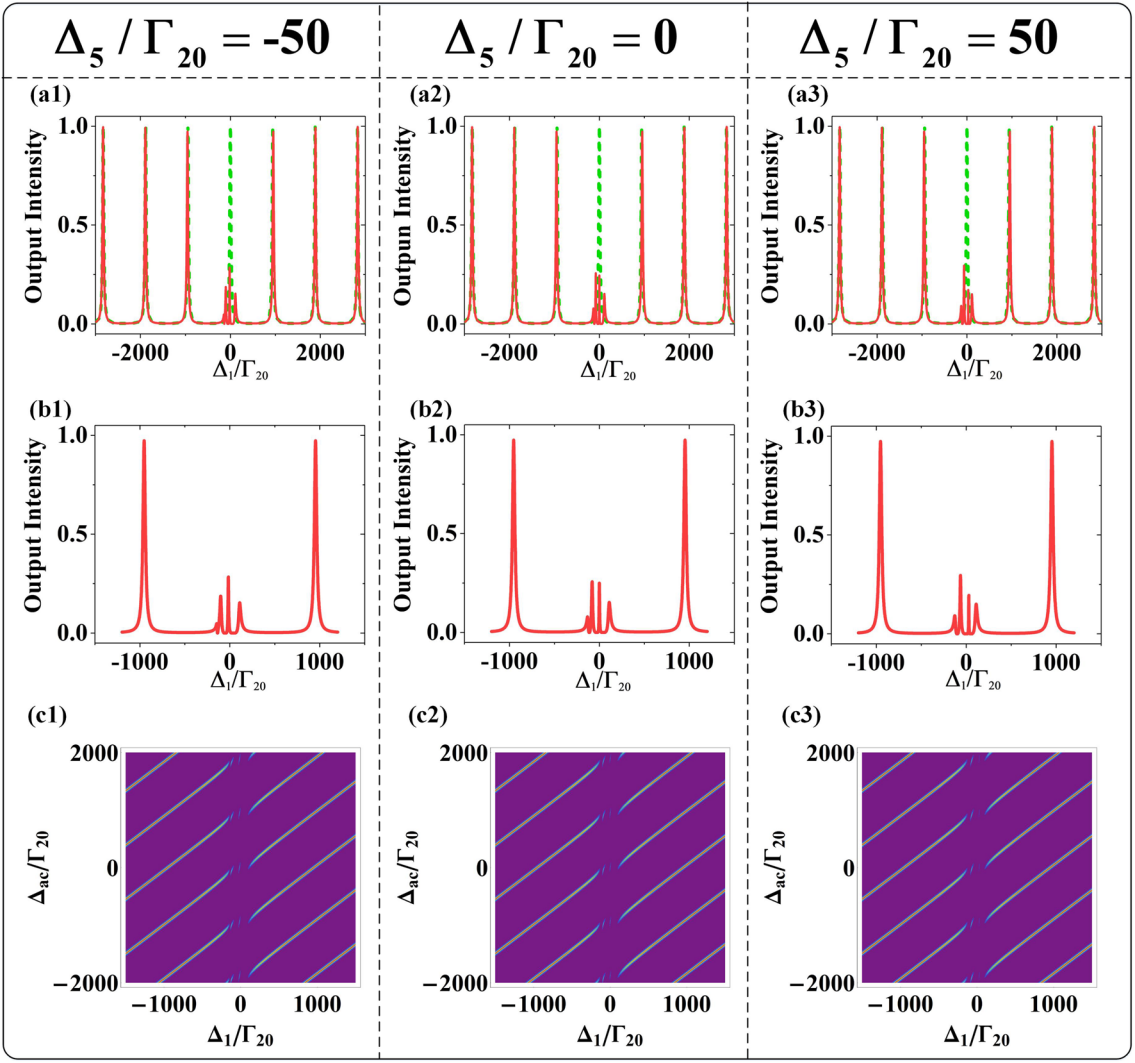
(**a1**)–(**a3**) The high-order modes intensity transmission coefficients of FWM signal of nested type at $\Delta_{ac} = 0\,{\text{MHz}}$. The solid curves represent the transmission peaks with different parameters. The dashed curves are the transmission spectra of empty cavity. (**a1**) With $\Delta_{5} /\Gamma_{20} = { - }50$. (**a2**) With $\Delta_{5} /\Gamma_{20} = 0$. (**a3**) With $\Delta_{5} /\Gamma_{20} = 50$. (**b1**)–(**b3**), The details of FWM intensity transmission coefficients in the narrow regions of high-order modes corresponding to (**a1**)–(**a3**). (**c1**)–(**c3**) The avoided-crossing plots with both scanning $\Delta_{1}$ and $\Delta_{ac}$ corresponding to (**a1**)–(**a3**).

First, we consider the influence of empty cavity on transmission FWM signal. The dashed lines in Fig. 4(a) show that the VRS peaks in high-order modes distribute evenly, which is equivalent to the free spectral region ($\Delta_{FSR}$). While the uniform distribution of the transmission peaks will be split when consider cavity field $g^{2} N$. Zero-order longitudinal mode $(m = 0)$ and high-order modes $(m = \pm 1, \pm 2, \pm {3} \ldots )$ can be split into dual-peaks at symmetrical center $\Delta_{1} = m\Delta_{FSR} /2$. The m-order transmission peaks are split into $m_{ \pm }$ with eigenvalues $\lambda_{m \pm }^{{(m,\Delta_{ac} )}} = - (\Delta_{ac} + m\Delta_{FSR} )/2 \pm \sqrt {(\Delta_{ac} + m\Delta_{FSR} )^{2} /4 + g^{2} N}$. Once probe field satisfies the enhancement condition $\Delta_{1}^{{(m,\Delta_{ac} )}} = - m\Delta_{FSR} - \lambda_{m \pm }^{{(m,\Delta_{ac} )}}$, the $m_{ \pm }$ splitting transmission peak are generated.

Next, we consider the splitting of the transmission FWM signal caused by the nested interaction in higher-order mode and we set up $E_{{2}}$ at non-resonant coupling condition $\Delta_{2} /\Gamma_{20} = 50\;{\text{}}$ to further study the nested three photon interaction. Figure 4(a1), (a2) and (a3) show four splitting zero-order modes under the existence of higher order cavity modes, which are equivalent to four transmission VRS peaks. Because of the indirect dressed effect of nested three-photons interaction, $E_{5}$ splits upper level $|2\rangle$ into dressed states $|2 \pm \rangle$ and produces two dressed channels. When probe field $E_{{1}}$ satisfies the four bright state conditions $\Delta {}_{{1}}{ + }\lambda_{{1 + }} { + }\lambda_{{1 + + }} = 0$, $\Delta {}_{{1}}{ + }\lambda_{{1 + }} { + }\lambda_{{1 + - }} = 0$, $\Delta {}_{{1}}{ + }\lambda_{{1{ - }}} { + }\lambda_{{1{ - + }}} = 0$ and $\Delta {}_{{1}}{ + }\lambda_{{1{ - }}} { + }\lambda_{{1{ - - }}} = 0$, four splitting modes are obtained. The tunable positions of these splitting modes can be achieved by controlling single factor variables $\Delta_{{5}} {/}\Gamma_{{{20}}} = - 50,0,50 {\text{}}$. Figure 4(b1) shows that whole of the four splitting modes move to negative region of scanning $\Delta_{1}$ because negative detuning $\Delta_{5} /\Gamma_{20} = - 50\;{\text{}}$ shifts up dressed states $|2 \pm \rangle$. In order to further study the distribution of splitting modes, the transmission FWM signal intensity as a function of detuning $\Delta_{1}$ and $\Delta_{ac}$ are investigate in Fig. 4(c). Figure 4(c1) shows that there are three peaks located at the negative region and the other peak locates at the positive region. In Fig. 4(b2), the positions of the four peaks are shifted to the negative region less shift than Fig. 4(b1) because zero detuning $\Delta_{{5}} = 0\,{\text{MHz}}$ can homogeneously distribute dressed states $|2 \pm \rangle$. What stands out in Fig. 4(c2) is that there is a dark state peak located at center scanning region $\Delta_{1} = 0\,{\text{MHz}}$. And other two peaks locate at negative region and last peak locates at positive region. The dark state peak can be obtained when $E_{2}$ drives dressed state $|2{ - }\rangle$ to resonantly couple with the virtual level $|{1}\rangle$, which obey the two-photon resonance condition $\Delta_{1} + \Delta_{2} + \lambda {}_{{2{ - }}} = 0$. When positive detuning $\Delta_{{5}} {/}\Gamma_{{{20}}} = 50$ shifts down dressed stated $|2 \pm \rangle$, the four peaks move to the positive region of scanning $\Delta_{1}$ in Fig. 4(b3), among which two locate at the negative region and the other two modes locate at the positive region shown in Fig. 4(c3). Briefly, $\Delta_{{5}}$ increases from a negative value to a positive value, and the positions of the four splitting modes shift from a negative region to a positive region.

### Splitting of high-order modes in the cascade type

Because dark resonance can form dark state FWM peak under cascade interaction, the zero-order mode should also split to form dark state mode. In order to confirm this idea, we set up a non-resonant coupling condition at $\Delta_{{2}} {/}\Gamma_{{{20}}} = 50$ to study whether dual two-photon cascade interaction can realize dark state peak. Similarly, we set up a single factor variable $\Delta_{3} /\Gamma_{20} = - 50,0,50$ to study the change of the number and position of mode splitting.

**Figure 5:**
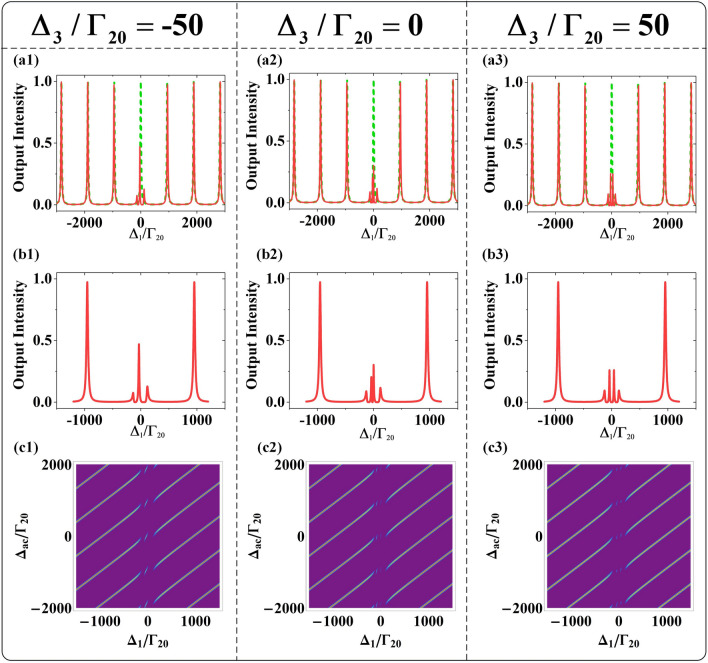
(**a1**)–(**a3**) The high-order modes intensity transmission coefficients of FWM signal of cascaded type at $\Delta_{ac} = 0\,{\text{MHz}}$. The solid curves represent the transmission peaks with different parameters. The dashed curves are the transmission spectra of empty cavities. (**a1**) With $\Delta_{3} /\Gamma_{20} = { - }50$. (**a2**) With $\Delta_{3} /\Gamma_{20} = 0$. (**a3**) With $\Delta_{3} /\Gamma_{20} = 50$. (**b1**)–(**b3**), The details of FWM intensity transmission coefficients in the narrow regions of high-order modes corresponding to (**a1**)–(**a3**). (**c1**)–(**c3**) The avoided-crossing plots with both scanning $\Delta_{1}$ and $\Delta_{ac}$ corresponding to (**a1**)–(**a3**).

The three splitting modes shown in Fig. 5(a1) because when $E{}_{2}$ and $E{}_{3}$ simultaneously split dressed state $|1 + \rangle$ to secondary dressed states $|1 + \pm \rangle$ at $\Delta_{3} /\Gamma_{20} = { - }50$, the probe field satisfies three bright state conditions $\Delta_{{1}} { + }\lambda_{1 + } + \lambda_{1 + + } = 0$, $\Delta_{{1}} { + }\lambda_{1 + } + \lambda_{{1 + - }} = 0$ and $\Delta_{{1}} { + }\lambda_{{1{ - }}} = 0$. Figure 5(b1) shows one of the three splitting modes with the highest intensity, which is due to the fact that the dressed energy level is closer to the virtual energy level $|1\rangle$ under the combined effect of $E{}_{2}$ and $E{}_{3}$. The details of the splitting modes variable location are shown in Fig. 5(c1) of transmission FWM signal intensity distribution as a function of detuning $\Delta_{1}$ and $\Delta_{ac}$, among which two modes locate at negative region and the other locates at positive region. In the case of $\Delta_{3} /\Gamma_{20} = 0$, the four splitting zero-order modes are realized in Fig. 5(a2). Three of the peaks are generated because probe field satisfies three bright conditions $\Delta_{{1}} { + }\lambda_{1 + } + \lambda_{1 + + } = 0$, $\Delta_{{1}} { + }\lambda_{1 + } + \lambda_{{1 + { - }}} = 0$ and $\Delta_{{1}} { + }\lambda_{{1{ - }}} = {0}$, and the other peak at the scanning center of $\Delta_{1}$ is a dark mode that satisfy the dark resonance condition $\Delta_{1} - \Delta_{3} = 0$. It is precisely because the dark state peak is generated at the resonance position of virtual level $|1\rangle$ that its intensity reaches the maximum shown in Fig. 5(b2). Figure 5(c2) presents that there are two peaks located at the negative region and the dark state peak located at $\Delta_{1} = 0\,{\text{MHz}}$ and the last peak at positive region. At condition $\Delta_{3} /\Gamma_{20} = 50$, Fig. 5(a3) displays four splitting modes symmetrically distribute along the scanning center of $\Delta_{1}$. When $E{}_{2}$ evenly splits state $|1 + \rangle$ into secondary dressed states $|1 + \pm \rangle$ and $E{}_{3}$ also evenly splits state $|1{ - }\rangle$ into secondary splitting states $|1{ - } \pm \rangle$, the four peaks will be symmetrically distributed as probe field satisfy the four bright conditions $\Delta_{{1}} { + }\lambda_{1 + } + \lambda_{1 + + } = 0$, $\Delta_{{1}} { + }\lambda_{1 + } + \lambda_{{1 + { - }}} = 0$, $\Delta_{{1}} { + }\lambda_{{1{ - }}} + \lambda_{{1{ - } + }} = 0$ and $\Delta_{{1}} { + }\lambda_{{1{ - }}} + \lambda_{{1{ - - }}} = 0$. Figure 5(b3) identifies the peak of equal intensity distribution in the center of scanning $\Delta_{1}$, which is caused by the uniform splitting of the dressed states. These peaks symmetrically located around the center $\Delta_{1} = 0\,{\text{MHz}}$ show in Fig. 5(c3), among which two peaks at negative region and the other two peaks at positive region. $\Delta_{{3}}$ changes from negative value to positive value, which not only increases the split modes, but also shifts the modes distribution from negative region to positive region.

## OB behaviors under VRS

Regarding the feedback effects of nested and cascade type, this section will study how the different interactions influence OB behavior^34^. As only the FWM signal circulates in the ring cavity while the probe field and other coupling fields do not, the FWM signal field is identified as a cavity mode. Therefore, the expression of transmission cavity FWM signal influenced by the feedback effect in nested type is adjusted as follows:

8 $$a_{Fn} \propto \frac{{ig\sqrt N G_{Fn} }}{{\left[ {d_{1}^{{\prime}} d_{3} (d_{1} )^{{\prime}} \left( {d_{1} + \frac{{g^{2} N}}{{d_{1}^{{\prime}} }} + \frac{{\left| {G_{2} } \right|^{2} }}{{d_{2} + \left| {G_{5} } \right|^{2} /d_{5} }} + \frac{{\left| {G_{Fn} } \right|^{2} }}{{\Gamma_{00} }}} \right)} \right]}}.$$

For the cascade type, the expression is given as follows:

9 $$a_{Fc} \propto \frac{{ig\sqrt N G_{Fc} }}{{\left[ {d_{1}^{{\prime}} d_{4} (d_{1} )^{{\prime}} \left( {d_{1} + \frac{{g^{2} N}}{{d_{1}^{{\prime}} }} + \frac{{\left| {G_{2} } \right|^{2} }}{{d_{2} }} + \frac{{\left| {G_{3} } \right|^{2} }}{{d_{3} }} + \frac{{\left| {G_{Fc} } \right|^{2} }}{{\Gamma_{00} }}} \right)} \right]}}.$$

On obtaining input intensity derived from the amplitude of probe field $E_{1}$ and the output intensity origin from the multicycle FWM signal field, the input and output relationship of OB behavior are produced in this system. The relationship expression for the nested type presents as follows:

10 $$\frac{{I_{on} }}{{I_{i} }} \propto \left| {ig\sqrt N G_{3} (G_{3}^{{\prime}} )^{2} [d_{1}^{{\prime}} d_{3} d_{1}^{2} (d_{1} + \frac{{g^{2} N}}{{d_{1}^{{\prime}} }} + \frac{{\left| {G_{2} } \right|^{2} }}{{d_{2} + \left| {G_{5} } \right|^{2} /d_{5} }} + \frac{{I_{on} }}{{\Gamma_{00} }})]^{ - 1} } \right|^{2} .$$

For the cascade type, the expression of input and output relationship is:

11 $$\frac{{I_{oc} }}{{I_{i} }} \propto \left| {ig\sqrt N G_{4} (G_{4}^{{\prime}} )^{2} [d_{1}^{{\prime}} d_{4} d_{1}^{2} (d_{1} + \frac{{g^{2} N}}{{d_{1}^{{\prime}} }} + \frac{{\left| {G_{2} } \right|^{2} }}{{d_{2} }} + \frac{{\left| {G_{3} } \right|^{2} }}{{d_{3} }} + \frac{{I_{oc} }}{{\Gamma_{00} }})]^{ - 1} } \right|^{2} .$$

**Figure 6:**
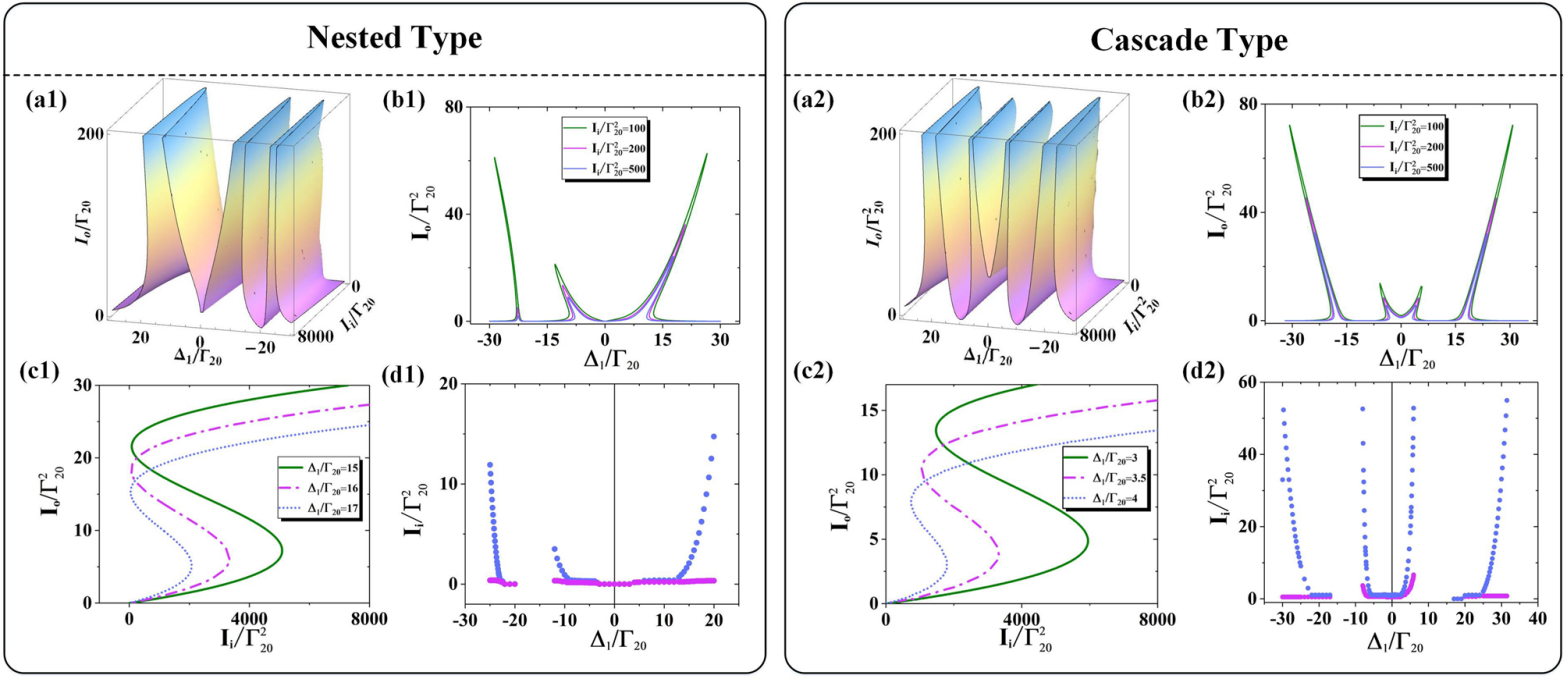
For nested type: (**a1**) The variation of transmission output intensity as function of $\Delta {}_{{1}}$ and input intensity $I_{i}$. (**b1**) The change trend of transmission output intensity with probe input intensity $I_{i} /\Gamma_{20}^{2} = 100,200,500$ from inside to outside. (**c1**) The variation relationship of transmission output intensity with detuning $\Delta_{1} /\Gamma_{20} = 15,16,17$ from right to left. (**d1**) The relationship of input intensity with detuning $\Delta_{1}$. Right OB threshold (blue dot), left OB threshold (purple dot). For cascaded type: (**a2**) The variation of transmission output intensity as function of $\Delta {}_{{1}}$ and input intensity $I_{i}$. (**b2**) With input intensity $I_{i} /\Gamma_{20}^{2} = 100,200,500$. (**c2**) With $\Delta_{1} /\Gamma_{20} = 3,3.5,4$. (**d2**) The relationship of input intensity with detuning $\Delta_{1}$. The other parameters are $G_{2} = 10\,{\text{MHz}}$, $G_{3} = 10\,{\text{MHz}}$, $\Delta_{2} /\Gamma_{20} = 10$, $\Delta_{3} /\Gamma_{20} = 10$, $\Delta_{ac} = 0\,{\text{MHz}}$, $G_{2} = 10\,{\text{MHz}}$, $G_{5} = 10\,{\text{MHz}}$, $\Delta_{2} /\Gamma_{20} = 10$, $\Delta_{5} = 0\,{\text{MHz}}$, $\Delta_{ac} = 0\,{\text{MHz}}$.

The first set of analyses examined the role of nested interaction in OB when the coupling fields $E_{2}$ and $E_{5}$ are both strong enough to lead the three-photon dressed effect. Figure 6(a1) shows that there are three split OB intensity peaks for satisfying the enhancement conditions $\Delta_{1} + \lambda_{1 + } + \lambda_{1 + \pm } = 0$ and $\Delta_{1} + \lambda_{{1{ - }}} = 0$, where the eigenvalues are $\lambda_{{{1 + } \pm }} = (\Delta_{{2}} + \lambda_{{{2} \pm }} - \lambda_{{1 + }} {)/2} \pm \sqrt {(\Delta_{2} + \lambda_{{{2} \pm }} - \lambda_{{1 + }} {)}^{2} {/4 + }\left| {G_{2} } \right|^{2} }$ and $\lambda_{2 \pm } = \Delta_{5} /2 \pm \sqrt {\Delta_{5}^{2} /2 + \left| {G_{5} } \right|^{2} }$. Figure 6(b1) reveals a positive correlation between output intensity and input intensity as well as a slight increase bend of output curves when $I{}_{i}$ increases from 100 to 200 and to 500. The inclined curves origin from the feedback effect of both three-photon dressed effects and cavity field $g^{2} N$. As $\Delta_{1}$ increases, the fast growth of right threshold of OB obtained in Fig. 6(c1). Figure 6(d1) demonstrates the enlarging OB scope with increased $\left| {\Delta_{1} } \right|$, where the right thresholds sharply increase with slight growth in the left threshold. Besides, we observed that there are two points of no OB behavior at or close to the points $\Delta_{1} = 0\,{\text{MHz}}$ and $\Delta_{1} /\Gamma_{20} = { - }20$, for obeying dark resonance conditions $\Delta_{1} + \Delta_{2} + \lambda_{2 + } = 0$ and $\Delta_{1} + \Delta_{2} + \lambda_{{2{ - }}} = 0$, respectively. The phenomenon of no OB resulted from the disappearance of dispersion effect induced by the nested three-photon interaction. In short, the OB behavior of three peaks and two points of no OB behavior distributed asymmetrically at the center of $\Delta_{1} = 0\,{\text{MHz}}$ under the nested interaction.

Next, we research the role of cascade interaction in OB and compare the difference with nested interaction. As the coupling fields $E_{2}$ and $E_{3}$ work as dual two-photon dressed effect, the symmetrical distribution of OB peaks at the center of $\Delta_{1} = 0\,{\text{MHz}}$ is obtained. Figure 6(a2) displays four split OB intensity peaks at symmetrical distribution. Similarly, the four peaks satisfy four enhancement conditions $\Delta_{1} + \lambda_{1 + } + \lambda_{1 + + } = 0$, $\Delta_{1} + \lambda_{1 + } + {\uplambda }_{{1 + - }} = 0$, $\Delta_{1} + \lambda_{{1{ - }}} + \lambda_{{1{ - } + }} = 0$ and $\Delta_{1} + \lambda_{{1{ - }}} + \lambda_{{1{ - - }}} = 0$, where the eigenvalues are $\lambda_{1 + \pm } = (\Delta_{{2}} - \lambda_{1 + } {)/2} \pm \sqrt {(\Delta_{2} - \lambda_{1 + } {)}^{2} {/4 + }\left| {G_{2} } \right|^{2} }$ and $\lambda_{{1{ - } \pm }} = - (\Delta_{{3}} { + }\lambda_{{1{ - }}} {)/2} \pm \sqrt {(\Delta_{3} { + }\lambda_{{1{ - }}} {)}^{2} {/4 + }\left| {G_{3} } \right|^{2} }$. Figure 6(b2) shows the positive correlation of the output intensity and input intensity, which is in the same situation of the nested type. Figure 6(c2) displays the strongly growth of right threshold as $\Delta_{1}$ increases. The changing relationship of output intensity with $\Delta_{1}$ is agree with the nested interaction. The obvious OB behavior contrasts with nested type is that there is an unchanged OB scope with similar growth trend of right threshold and left threshold presented in Fig. 6(d2), and there are another two broadened OB scopes with fast growth of right thresholds and slowly changed left thresholds. In addition, the two points of no OB located around the posits of $\Delta_{{1}}/\Gamma_{{{20}}} = -{10},{{10}}$ obey dark resonance conditions $\Delta_{1} + \Delta_{2} = 0$ and $\Delta_{1} - \Delta_{3} = 0$, which resulted from the cascaded dual two-phonon interaction and leaded the disappearances of dispersion effect. The OB behavior under the work of cascaded interaction with four peaks and two points of  OB distributed symmetrically at the center of $\Delta_{1} = 0\,{\text{MHz}}$. That is to say, the indirect dressed effect of $E_{5}$ in nested interaction and direct dressed effect of $E_{3}$ in the cascaded interaction lead to the different distribution of OB.

## Conclusion

This study developed the type of interaction between laser and atom in a ring resonator, named as nested interaction and cascaded interaction, and studies the influence of these two nonlinear processes on VRS and OB. The research showed that the quantities of VRS can be adjusted to be four in three-photon nested interaction and three in dual two-photon cascaded interaction, and revealed that dark resonance determines the number of VRS. The study also investigated the position modulation of VRS under high-order modes as well as the controllable intensity of splitting peak. Moreover, we analysis the effect of dark resonances in OB behavior and observed asymmetrical distribution in the nested type with fast growth right thresholds and unchanged left thresholds, contrasting with the symmetric distribution in the cascade type with additional same increased trend of both right and left thresholds. Taken together, VRS and OB can be regulated by dark states under different interactions, so that they can be widely used in the field of dark state limitation, such as all-optical quantum logic devices, optical switch, optical memory, optical limiting, optical oscillation, and nonlinear optical devices.
